# Tumor Suppressive Function of mir-205 in Breast Cancer Is Linked to HMGB3 Regulation

**DOI:** 10.1371/journal.pone.0076402

**Published:** 2013-10-02

**Authors:** Ola A. Elgamal, Jong-Kook Park, Yuriy Gusev, Ana Clara P. Azevedo-Pouly, Jinmai Jiang, Avtar Roopra, Thomas D. Schmittgen

**Affiliations:** 1 College of Pharmacy, the Ohio State University, Columbus, Ohio, United States of America; 2 Georgetown University Cancer Center, Washington, District of Columbia, United States of America; 3 Department of Neuroscience, University of Wisconsin, Madison, Wisconsin, United States of America; The University of Hong Kong, China

## Abstract

Identifying targets of dysregulated microRNAs (miRNAs) will enhance our understanding of how altered miRNA expression contributes to the malignant phenotype of breast cancer. The expression of miR-205 was reduced in four breast cancer cell lines compared to the normal-like epithelial cell line MCF10A and in tumor and metastatic tissues compared to adjacent benign breast tissue. Two predicted binding sites for miR-205 were identified in the 3’ untranslated region of the high mobility group box 3 gene, HMGB3. Both dual-luciferase reporter assay and Western blotting confirmed that miR-205 binds to and regulates HMGB3. To further explore miR-205 targeting of HMGB3, WST-1 proliferation and in vitro invasion assays were performed in MDA-MB-231 and BT549 cells transiently transfected with precursor miR-205 oligonucleotide or HMGB3 small interfering RNA (siRNA). Both treatments reduced the proliferation and invasion of the cancer cells. The mRNA and protein levels of HMGB3 were higher in the tumor compared to adjacent benign specimens and there was an indirect correlation between the expression of HMGB3 mRNA and patient survival. Treatment of breast cancer cells with 5-Aza/TSA derepressed miR-205 and reduced HMGB3 mRNA while knockdown of the transcriptional repressor NRSF/REST, reduced miR-205 and increased HMGB3. In conclusion, regulation of HMGB3 by miR-205 reduced both proliferation and invasion of breast cancer cells. Our findings suggest that modulating miR-205 and/or targeting HMGB3 are potential therapies for advanced breast cancer.

## Introduction

Breast cancer is the second most prevalent cancer in the USA and is the second most common cause of cancer-related death in this country [1]. It is estimated that 230,480 women will develop breast cancer in 2011 and 39,520 will die of this disease [2]. Like many cancers, breast cancer is a heterogeneous disease that differs molecularly, biologically and clinically. Breast cancer is commonly classified by the estrogen/progesterone (ER/PR) receptor status and the HER2 (ERBB2) amplification status. Breast cancers that are ER+ and or PR+ are treatable with hormonal therapies such as Tamoxifen, while patients having HER2 amplification respond to receptor tyrosine kinase inhibitors such as Trastuzumab. Triple-negative breast cancer (TNBC) is defined by the absence of ER and PR expression and HER2 amplification. Approximately 10-20% of breast cancers are TNBC [3]. These patients have the worse prognosis because of the lack of effective targeted therapeutics [4]. The molecular basis for the TNBC is poorly understood. Successful treatments for TNBC may be possible once a more fundamental understanding of the aggressive phenotype is understood.

Expression of the microRNA, miR-205 is enriched in esophagus, trachea, breast, thymus and prostate tissues [5]. miR-205 is deregulated in many solid tumors; the expression is increased in some tumors while it is decreased in others. miR-205 was overexpressed in endometrial cancer [6] and non-small cell lung cancer [7-9], while its’ expression was downregulated in prostate cancer [10], melanoma [11-13] and breast cancers [14,15]. miR-205 is found exclusively in normal ducts and lobular myoepithelial cells of the breast but is significantly reduced in breast tumor tissues [15,16]. The purported tumor suppressive functions of miR-205 in breast cancer is due to direct targeting of several oncogenes such as VEGFA, E2F1, E2F5, PKC epsilon and HER3 reviewed in 16 as well as attenuating epithelial to mesenchymal transition (EMT) by suppressing ZEB1 and ZEB2 [16-18].

The intent of this study was to identify additional target genes for miR-205 that may be involved in the aggressive phenotype of TNBC. We identified HMGB3, a member of the high mobility group protein superfamily. We show that increased expression of HMGB3, due to reduced miR-205 expression, causes increased cell proliferation and in vitro invasion. Furthermore, breast cancer patients with increased HMGB3 expression have worse survival. These findings suggest that HMGB3 may serve as a biomarker and/or therapeutic target for breast cancer.

## Materials and Methods

### Ethics Statement

All research involving human specimens has been approved by the Ohio State University institutional review board.

### Cell lines

MCF10A, MCF7, MDA-MB-231, MDA-MB-436 and BT549 cell lines were obtained from the American Type Culture Collection or were supplied by various investigators. MCF-10A cells stably expressing REST shRNA were generated as described [19]. MCF10A was cultured in DMEM and F-12 (1:1 ratio) containing 5% horse serum, hydrocortisone, human EGF, cholera toxin, insulin and 1% penicillin and streptomycin as described [19]. MCF7 and BT549 cell lines were maintained in MEM media supplemented with 10% FBS. MDA-MB-231 and MDA-MB-436 cells were cultured in DMEM with 10% FBS. All cell lines were cultured under standard conditions.

### Tissue procurement

Snap frozen specimens of 33 human breast tissues were supplied by the Midwest division of the Cooperative Human Tissue Network (Columbus, OH). These included 11 matched pairs of tumor and normal adjacent tissue as well as metastatic disease. Clinical data on these tissues are provided in Table S1.

### RNA extraction

Total RNA was extracted from the cell lines and tissues using Trizol (Invitrogen) and RNeasy mini kit (Qiagen), respectively, according to the manufacturer’s instructions. Integrity of the RNA was measured by the Agilent Bioanlyzer; RIN (RNA integrity number) of 7 or higher were considered to have satisfactory integrity.

### Real-time PCR

Mature miR-205-5p levels were assayed using TaqMan microRNA Reverse Transcription kit and TaqMan MicroRNA Assay (Applied Biosystems) per the manufacturer’s instructions. Data were normalized to 18S rRNA and the relative gene expression was calculated using the comparative C_T_ method as described [20]. Primers used in the qRT-PCR are provided in Table S2.

### Transient transfection assays

For HMGB3 functional studies, MDA-MB-231 and BT549 cell lines were lipofectamine 2000 transfected (Invitrogen) with HMGB3 siRNA or control siRNA (Dharmacon). Target identification studies were conducted by transfecting the same cell lines with precursor miR-205-5p mimic oligonucleotides (pre-miR-205-5p) or pre-miR negative control (Dharmacon). Transient transfection assays were conducted for 48 h with 100 nM of oligonucleotides.

### miR-205 overexpressing stable cells

Partial length of the miR-205 primary precursor sequence was cloned and inserted into the pcDNA3.1(+) vector. MDA-MB-231 cells were transfected with the construct and selected using G418, Geneticin^®^.

### Lentiviral knockdown of REST in MCF7 cells

Stable REST knockdown in MCF7 cells was achieved using a Dharmacon SMARTvector lentiviral shRNA delivery system as per manufacturer’s instructions. Briefly, cells were infected in the presence of 8 mg/mL polybrene at an MOI of 5 with virus expressing a non- targeting control or REST shRNA. Puromycin selection was initiated 48 h after infection and maintained during cell expansion. SMARTvector Lentiviral Particles (catalog # SH-042194-01-25) towards REST targeted the sequence GCAAACACCTCAATCGCCA, Non-Targeting SMARTvector shRNA Lentiviral particles (catalog # S-005000- 01) were used as an infection control.

### Luciferase reporter assay

Approximately 2.8 kbp of the HMGB3 3’ untranslated region (3’ UTR) containing two predicted miR-205 binding sites was PCR amplified and inserted downstream of the Renilla luciferase gene in the PsiCheck-2 plasmid (Promega). MDA-MB-231 cells were cotransfected with the HMGB3 PsiCheck-2 construct and 100 nM of pre-mir-205 or control oligo. After 48 h, the Renilla and Firefly luciferase activities were measured using the Dual-Luciferase Reporter Assay kit (Promega).

### Western Blotting and antibodies

Protein was extracted from cell lines and human tissues using standard conditions. Anti-HMGB3 antibody (Abgent, # AJ1365) and anti-GAPDH (SC-32233, Santa Cruz Biotechnology).

### WST-1 proliferation assay

Cells were plated in 96-well plate overnight and then transfected with 100 nM oligos. Following 96 h, WST-1 reagent (Roche) was added for 1.5 h and absorbance was measured using standard conditions.

### Matrigel invasion assay

Cells (2×10^5^) were plated in 60 mm dishes. Following an overnight incubation, they were transfected with 100 nM oligonucleotides. After 48 h, the cells were collected and 5×10^4^ cells were seeded onto Matrigel coated inserts (3µm pore size, BD Biosciences) for 24 h. Cells that migrated through the Matrigel were visualized following fixation with methanol and staining with crystal violet.

### Immunohistochemistry

Human breast tissues were fixed overnight in 10% formalin and then stained for HMGB3 using antibody (Abgent, # AJ1365) using standard techniques.

### Drug treatment

In a 6-well plate, 2×10^5^ cells were plated per well. Following an overnight incubation, the cells were exposed to 10 µM 5-Aza-2’-Deoxycytidine (5-Aza) (InSolution™ Millipore), 600 nM TSA (Trichostatin A, Sigma) or a combination of both. The cells were exposed to 5-Aza for 96 h; fresh drug was added after 48 h. For the combination treatment, TSA was added on the third day and cells were collected on fourth day.

### Kaplan Meier survival plot

A disease free survival for breast cancer patients was analyzed as a function of HMGB3 gene expression using open access web resource G-DOC developed at Georgetown University Medical Center. See Methods S1 for detail. 

## Results

### miR-205 expression in breast cell lines and tissues

**Figure 1:**
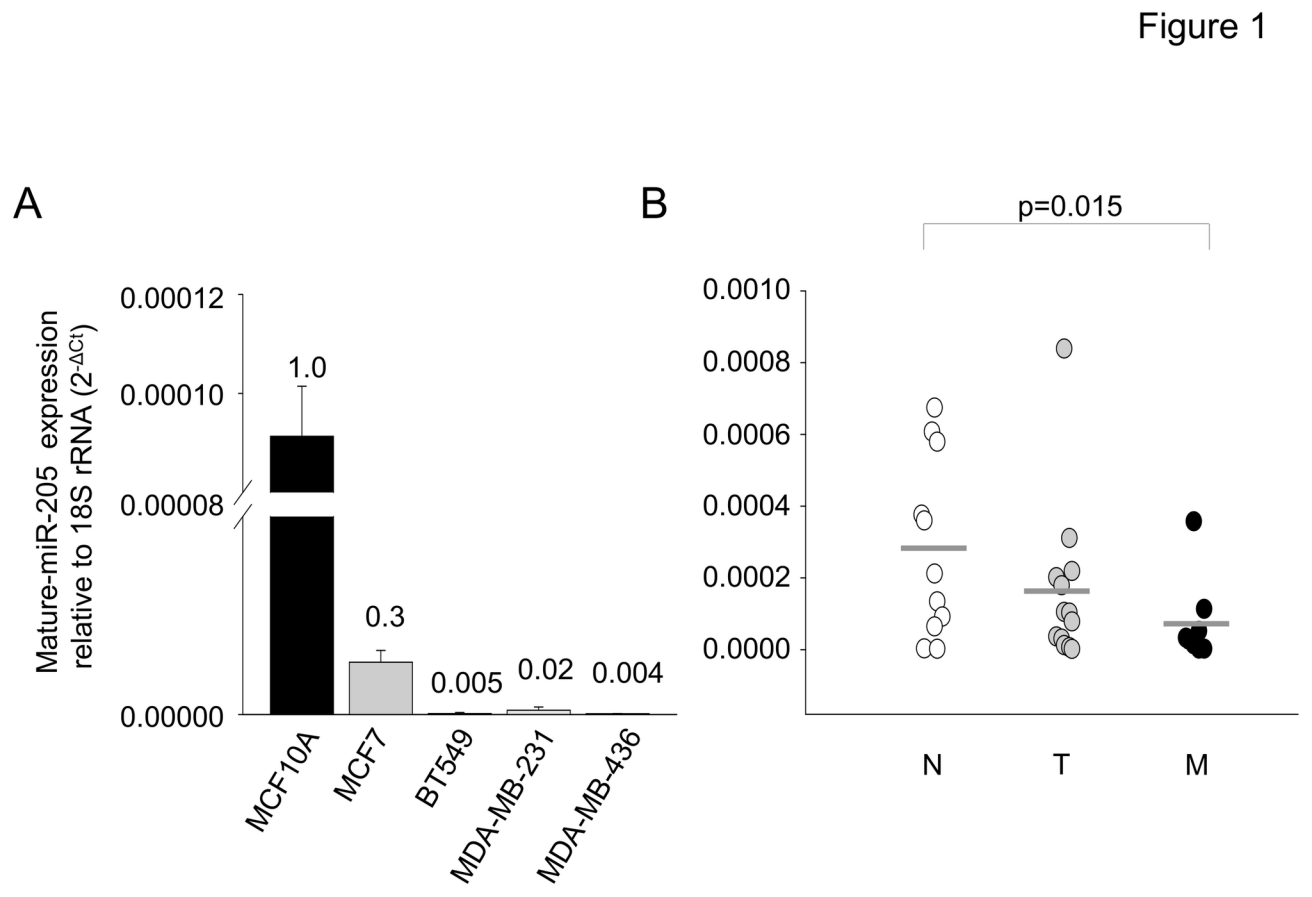
miR-205 is down-regulated in breast cancer. (A) Expression pattern of miR-205-5p in MCA10A, MCF7, BT549, MDA-MB-231, and MDA-MB-436 cells. The data in cancer cell lines are expressed as fold-change compared to MCA10A, which was assigned a value of “1”. (B) miR-205-5p levels in the normal adjacent breast tissues (N), non-metastatic tumor (T), and metastatic breast cancer specimens (M) were determined by qPCR. Data are presented relative to 18S rRNA. Mean values are indicated by horizontal bars.

The levels of miR-205 were measured in five breast cell lines. miR-205 was significantly reduced in BT549, MDA-MB-231 and MDA-MB-436 compared to the normal-like MCF10A breast cell line (Figure 1A). MCF7 is an ER/PR positive breast cancer cell line while BT549, MDA-MB-231 and MDA-MB-436 are TNBC cell lines. The levels of miR-205 were undetectable (C_T_ > 37) in the TNBC cell lines. miR-205 was measured in 33 human breast tissues by qRT-PCR. There was a trend of reduced miR-205 expression when progressing from the normal adjacent tissue to non-metastatic and metastatic disease (Figure 1B). These results are in accordance with previous reports about the significant reduction of miR-205 in TNBC compared to other types of breast cancer [21,22].

### Tumor suppressive role of miR-205

**Figure 2:**
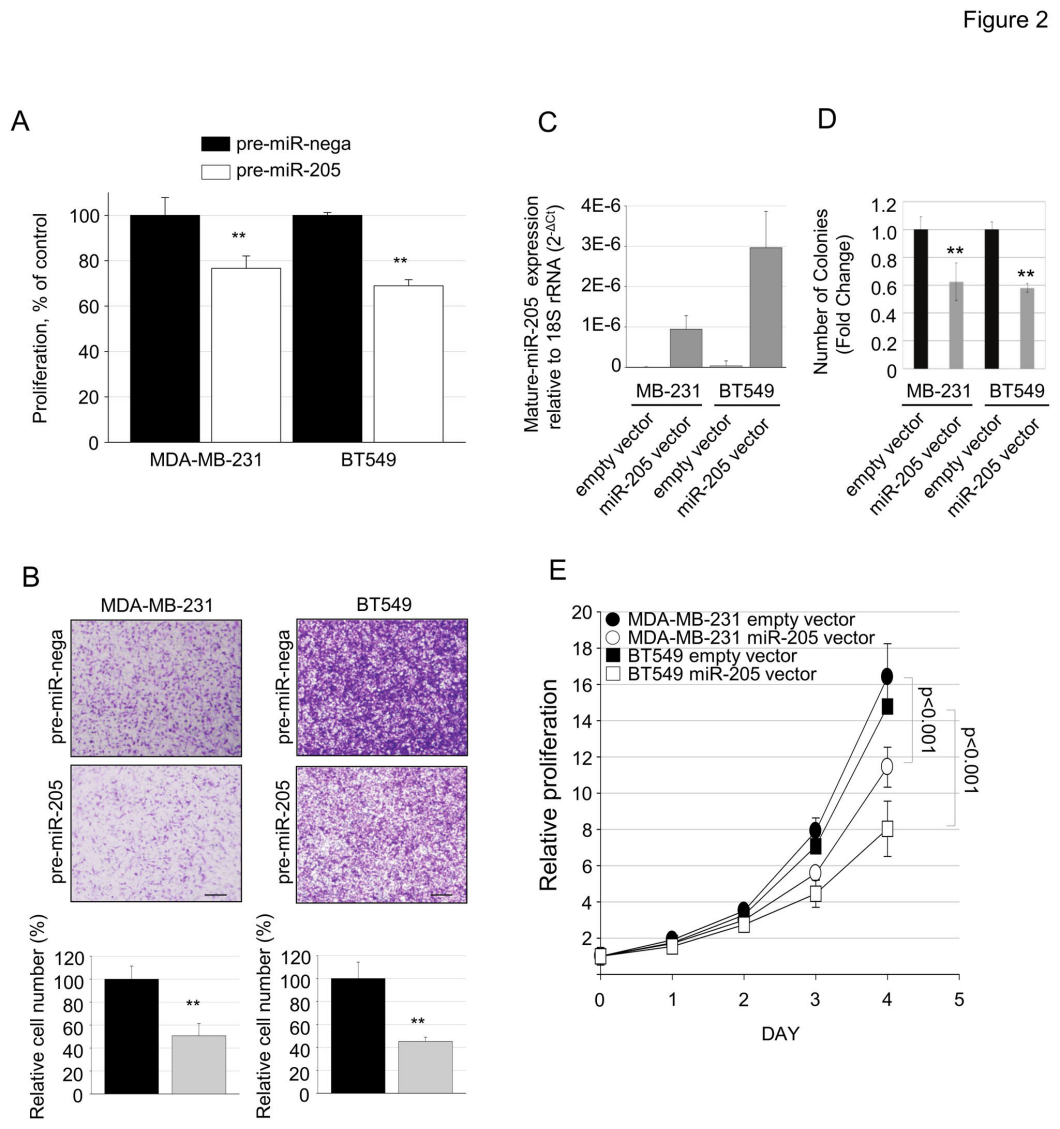
Over-expression of miR-205 reduces the proliferation and in vitro invasion of breast cancer cell lines. (A) MDA-MB-231 and BT549 cells were transfected with 100 nM of pre-miR-205 or control oligonucleotides. Following a 96 h exposure, cell proliferation was measured using a WST-1 assay. (B) Representative photographs showing in vitro Matrigel invasion assay of MDA-MB-231 and BT549 cells after transfection with pre-miR-control or pre-miR-205-5p 48 hr prior to seed into the upper chamber of 24-well transwell units. Bar graphs represent the mean±SD values of the relative number of invasive cells (n=4). (C) Real time TaqMan analysis of miR-205 levels in stable cell lines over-expressing primary transcripts of miR-205. (D) Relative representation of the number of colonies in MDA-MB-231 and BT549 cells stably expressing miR-205-5p. (E) Overall proliferation of miR-205-5p stably expressing MDA-MB-231 and BT549 cells up to 96 hrs.

Functional studies of miR-205 were conducted in MDA-MB-231 and BT549 TNBC cell lines using both transient transfection of pre-miR-205 oligo and stable miR-205 over expression. Cell proliferation (Figure 2A) and in vitro invasion (Figure 2B) of MDA-MB-231 and BT549 cells decreased following pre-miR-205 oligo transfection. Stable over expression of miR-205 in MDA-MB-231 and BT549 cells was confirmed by qRT-PCR (Figure 2C). Stable expression of pre-miR-205 reduced colony formation (Figure 2D) and proliferation (Figure 2E) of MDA-MB-231 and BT549 cells. These functional studies support the tumor suppressive role of miR-205 in breast cancer as reported by others [15].

### miR-205 targets HMGB3

**Figure 3:**
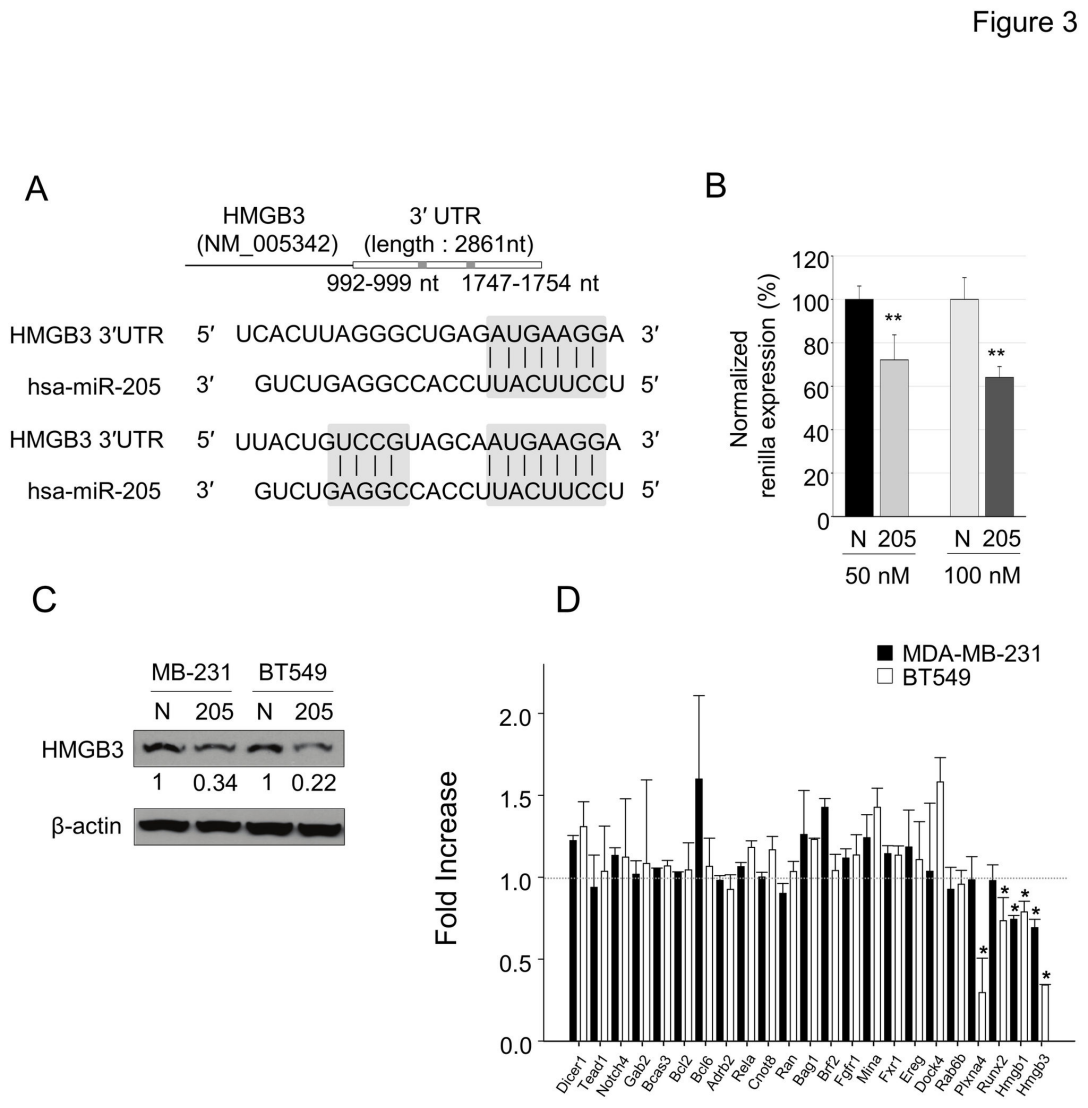
HMGB3 is a target of miR-205 in breast cancer. (A) Schematic diagram of miR-205 binding sites in the 3' UTR region of HMGB3 mRNA. (B) Luciferase reporter plasmids carrying the full length of HMGB3 3' UTR were transiently co-transfected with the negative control of pre-miR precursor (N) or the miR-205 precursor (205) at 50 and 100 nM concentrations. Luciferase activity was measured 24 h after transfection. The data are the mean ± SD of at least 3 independent transfections. (C) Lysates were immunoblotted for expression levels of HMGB3 in MDA-MB-231 and BT549 cells. Normalized expression to β-actin is included. (D) Expression of the selected predicted oncogenic target genes of miR-205 was evaluated in both MDA-MB-231 and BT549 cells 48 h after transfection of either control oligonucleotides or pre-miR-205-5p. Expression levels were calculated relative to 18S rRNA and the data are expressed as fold-increase compared to cells treated with control oligo (assigned a value of “1”).

To investigate novel targets, we noted that TargetScan predicted two miR-205 binding sites in the 3’ UTR of human HMGB3 (Figure 3A). Binding of miR-205 to the 3’ UTR of HMGB3 was validated using luciferase reporter assays (Figure 3B). HMGB3 protein was reduced in MDA-MB-231 and BT549 cells transfected with pre-miR-205 oligo compared to control oligo (Figure 3C). In an effort to identify other predicted targets of miR-205, we measured their mRNA expression in MDA-MB-231 and BT549 cell lines following transfection of pre-miR-205 or pre-miRNA control oligos. Of the 23 genes measured by qRT-PCR, 6 (DOCK4, RAB6B, PLXNA4, RUNX2, HMGB1 and HMGB3) were significantly reduced in the miR-205 transfected cells (Figure 3D). These data confirm that the reduction in HMGB3 protein by miR-205 results from a decrease in the HMGB3 mRNA and identifies a number of potential miR-205 target genes.

### Functional effects of miR-205/HMGB3 regulation in breast cancer

**Figure 4:**
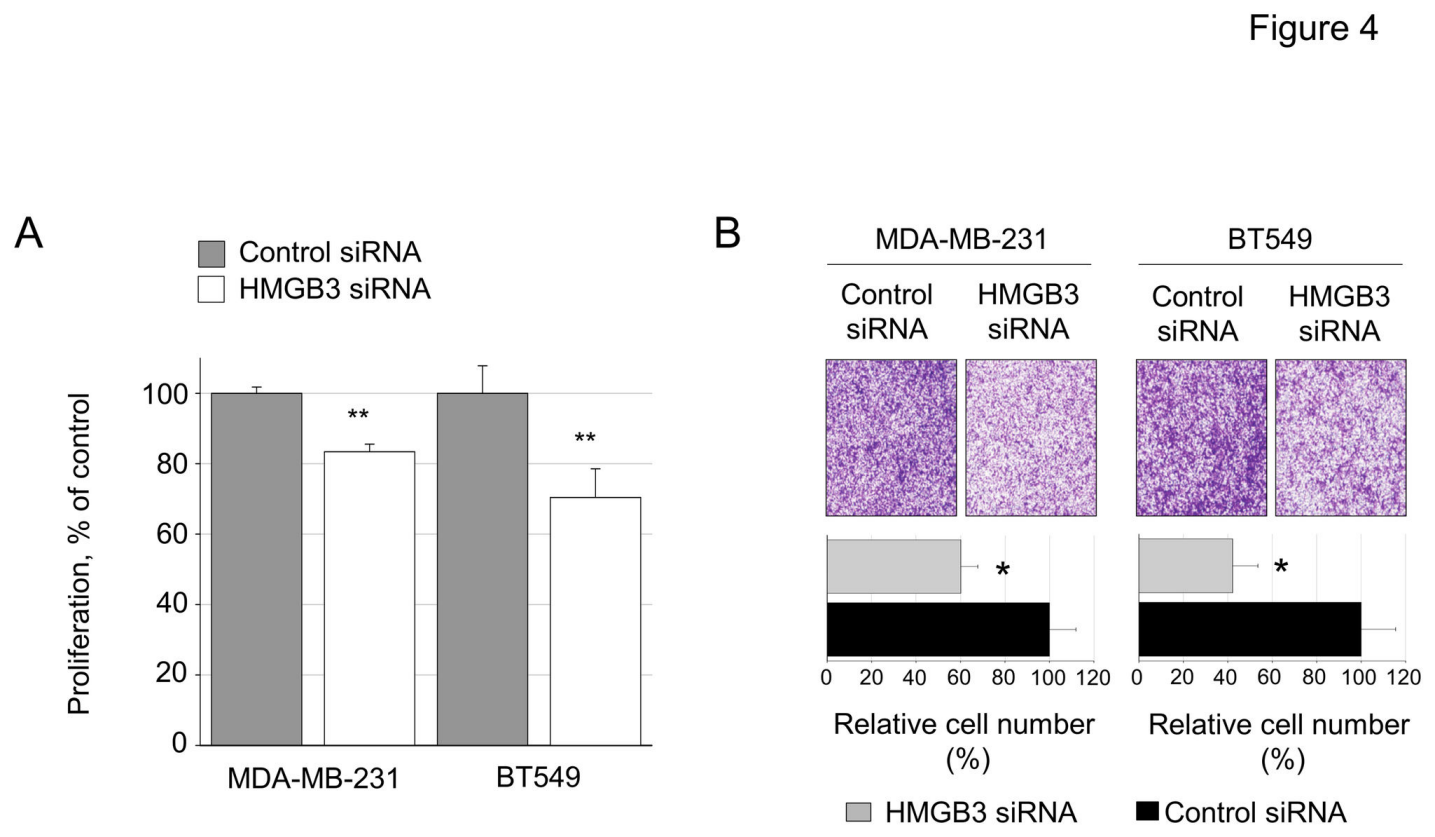
Knockdown of HMGB3 reduces the proliferation and in vitro invasion of breast cancer cells. (A) Proliferation of MDA-MB-231 and BT549 cells transfected with HMGB3 siRNA or control siRNA. (B) in vitro Matrigel invasion assay of MDA-MB-231 and BT549 cells after transfection with HMGB siRNA or control siRNA 48 hr prior to seed into the upper chamber of 24-well transwell units. Bar graphs represent the mean ± SD values of the relative number of invasive cells.

**Figure 5:**
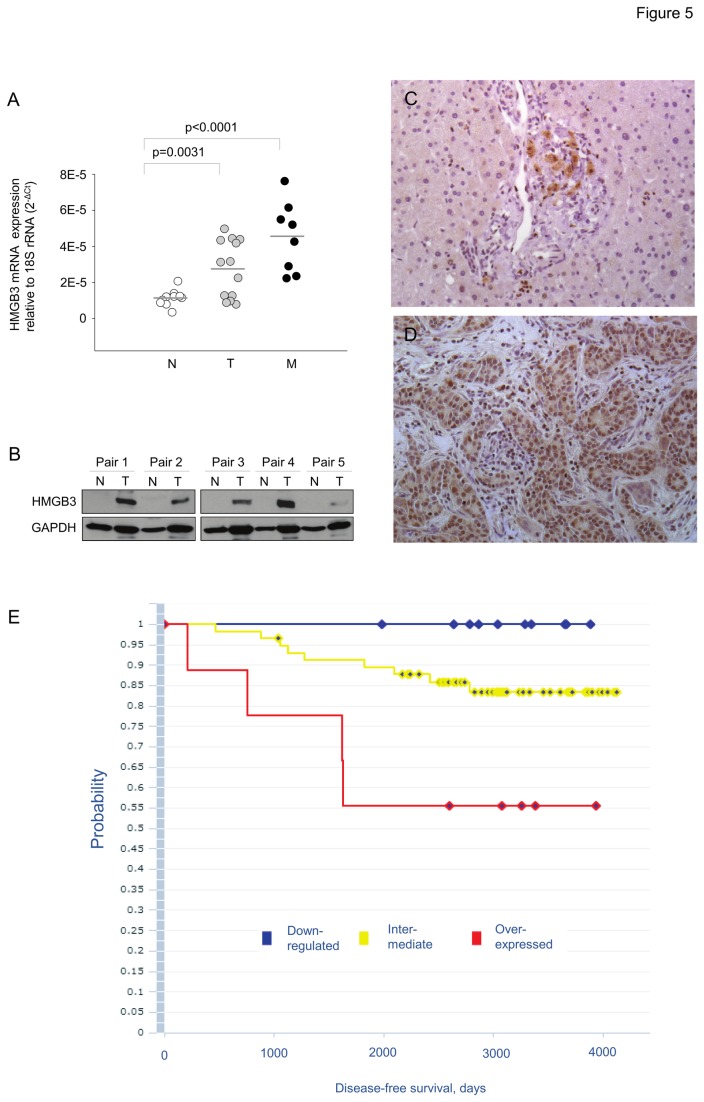
HMGB3 is over-expressed in primary breast cancer tissues. (A) Expression pattern of HMGB3 mRNA level in normal adjacent (N), non-metastatic tumor (T) and metastatic (M) breast cancer tissues. mRNA expression levels were calculated relative to 18S rRNA. (B) HMGB3 and GAPDH protein levels in non-metastatic and metastatic breast cancer compared to paired normal tissues was determined by western blotting. HMGB3 expression was confirmed by immunohistochemistry staining in human breast tissues. Brown color indicates HMGB3 staining in adjacent benign tissue (C) and tumor (D). (E) Kaplan Meier plot of disease free survival based on HMGB3 expression in breast cancer samples. Blue, patients with gene down-regulated by more than 1.2 fold; red, patients with genes overexpressed by more than 1.2 fold; yellow patients with intermediate values of fold change. P-values of log-rank test for each pair of KM curves are provided at the bottom of the plot.

To determine functions of HMGB3 overexpression in breast cancer, we conducted WST-1 proliferation and in vitro invasion assays in breast cancer cell lines. We found a significant reduction of proliferation (Figure 4A) and invasion (Figure 4B) of MDA-MB-231 and BT549 cells treated with HMGB3 siRNA compared to scrambled control siRNA. Thus the effects of suppressing HMGB3 mRNA either by miR-205 oligo or HMGB3 siRNA are identical in these cell lines (Figures 2, 4). The expression of HMGB3 mRNA was quantified in the 33 human breast tissues and was elevated in the metastatic group and in the breast tumors compared to unaffected adjacent benign tissue (Figure 5A). The HMGB3 protein levels were assayed in 5 matched pairs of the human breast tissues. HMGB3 was undetected in the normal adjacent tissue samples while abundantly present in the tumor tissue (Figure 5B). To identify tissue localization, we performed immunohistochemistry staining against HMGB3 protein and found that HMGB3 is localized in the glands and ducts of normal adjacent tissue while widespread in tumor tissue (Figure 5 C, D). To determine if a correlation exists between HMGB3 expression and patient survival, we analyzed published breast cancer data sets. Three groups of patients were identified, those with an intermediate tumor levels of HMGB3 and those with increased or decreased HMGB3 expression in the tumors. Those patients with over expressed HMGB3 expression had the poorest survival (P<0.05). Patients with down regulated HMGB3 expression survived longer than those with intermediate expression, however the difference was not significant (p=0.09) (Figure 5E).

### Relationship between miR-205, HMGB3 and EMT

Since miR-205 directly targets ZEB1 and ZEB2 in Madin Darby canine kidney cells [17], reduced expression of miR-205 could maintain breast cancer cells in the mesenchymal state through ZEB1/ZEB2 inhibition of E-cadherin (CDH1). We validated these findings in the TNBC cell lines MDA-MB-231 and BT549. These cell lines are considered to be mesenchymal-like [23]. miR-205 transfection decreased the protein levels of the mesenchymal N-cadherin, vimentin and ZEB1 (Figure S1). To relate HMGB3 to EMT, we measured epithelial and mesenchymal markers in MDA-MB-231 cells exposed to HMGB3 siRNA. Knocking down HMGB3 increased the expression of CDH1 while ZEB1 was essentially unchanged (Figure S2). Thus HMGB3 promotes EMT, however it is not through inhibition of ZEB1 protein levels (Figure S2).

### Chromatin modifying agents attenuate HMGB3 levels through derepression of miR-205

**Figure 6:**
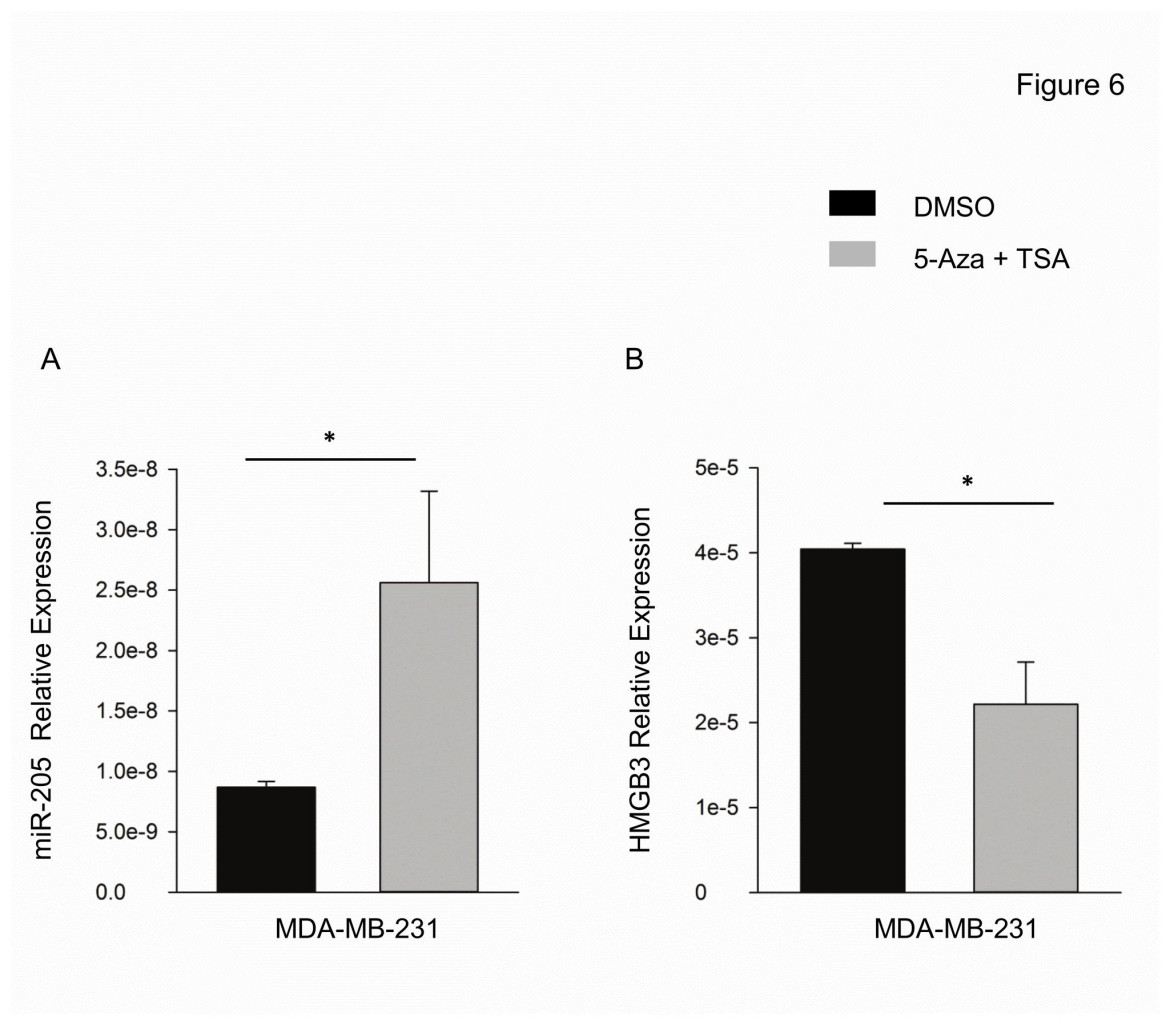
Expression of miR-205 and HMGB3 are altered in 5-Aza and TSA treated cells. (A) Increased miR-205 and (B) reduced HMGB3 expression in MDA-MB-231 cells treated with a combination of 5-Aza and TSA as described in Methods.

Repression of miR-205 in prostate and breast cancer cell lines has been linked to promotor hypermethylation of the miR-205 host gene LOC642587 [24]. Also H3K9 deacetylation contributed to the miR-205 suppression in prostate cancer cell lines [24]. Attempts as miR-205 derepression in TNBC cells were done by treating MDA-MB-231 cells with 5-Aza/TSA. 5-Aza/TSA treatment increased the miR-205 expression by more than 2-fold (Figure 6A) and decreased HMGB3 mRNA nearly 2-fold (Figure 6B). Thus small molecule treatment of breast cancer may impact HMGB3 via derepression of miR-205.

### Relationship between REST, miR-205 and HMGB3

**Figure 7:**
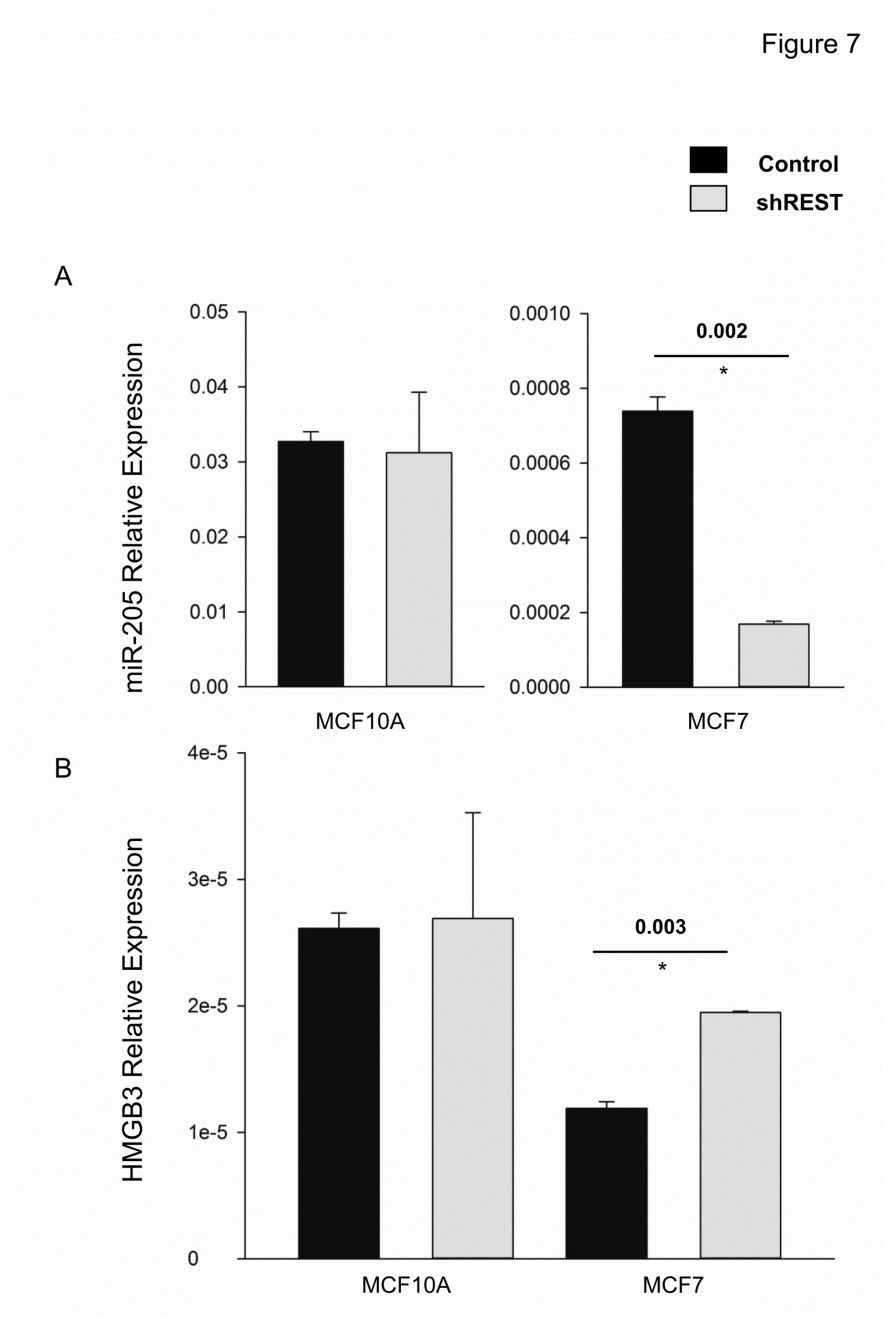
Change in miR-205 and HMGB3 expression in REST-less cells. (A) The amount of miR-205 in MCF7 cells stably expressing shRNA to REST (RESTless cells) was decreased compared to control but was unchanged in the RESTless MCF10A cell line. (B) HMGB3 mRNA levels increased only in the RESTless MCF7 cell line.

REST/NRSF is transcriptional repressor of neuronal genes in non-neuronal tissues. A subset of breast cancers with reduced expression of REST (i.e. REST-less tumors) display a highly aggressive phenotype that includes poor prognosis and increased likelihood of disease recurrence within the first 3 years after diagnosis [19]. We examined the miR-205 expression in REST-less MCF-7 and MCF-10A cells (i.e. cells stably expressing REST shRNA). REST knockdown significantly reduced the miR-205 expression in MCF-7 but not MCF-10A cells (Figure 7A, B). Concomitantly, HMGB3 levels had a significant increase by REST knockdown in MCF-7 but not MCF-10A cells (Figure 7 C, D). Thus, knockdown of REST enhanced the HMGB3 up-regulation, presumably through reduced miR-205, in the cancerous MCF-7 cells but had no effect on MCF-10A cells supporting the hypothesis that REST-less tumor cells display an aggressive phenotype [19].

## Discussion

The overall aim of this study is to support the tumor suppressor role of miR-205 in breast cancer and demonstrate that reintroduction or derepression of the miRNA in TNBC cell lines is beneficial. We report that the expression of HMGB3, a member of the high mobility group protein superfamily, is increased in advanced breast cancer. A link between HMGB3 and the invasive, metastatic phenotype has been made by demonstrating that HMGB3 mRNA levels are increased in metastatic breast cancers (Figure 5A), that knockdown of HMGB3 decreases in vitro invasiveness (Figure 4B) and patients with increased HMGB3 expression have poor survival (Figure 5E). Moreover, we link the up-regulation of HMGB3 in breast cancer to miR-205, a miRNA that has been shown here (Figure 1) and in other studies [14-16,18] to have reduced expression in breast cancer.

The HMG superfamily of DNA binding proteins recognize the HMG box, typically located in promoters. HMG proteins are referred to as architectural transcription factors [25]. The HMG family binds to and distorts DNA causing chromatin modification and facilitating either activating or repressing transcription factors to bind to the promoter [26]. There are two major subclasses of HMG proteins, HMGA and HMGB. There is structural resemblance between HMGB1, HMGB2 and HMGB3 which indicates similar biological functions for these proteins. Of the 4 HMGB proteins, HMGB1 has been the most heavily studied. HMGB1 is involved in many biological processes including DNA repair reviewed in 25. Previous studies have shown the oncogenic role of HMGB1 in cancer. When HMGB1 binds to its’ receptor RAGE (receptor for advanced glycation end-products) it promotes tumor growth and metastasis [27].

HMGB3 is present mainly in the bone marrow and is involved in hematopoietic stem cell renewal [28]. Recently, HMGB3 was reported to be a target for miR-206 and is involved in muscle regeneration [29]. It was among a group of embryonic stem cell-like transcriptional regulators overexpressed in poorly differentiated, high-grade tumors [30]. With the exception of the work by Ben-Porath, et al. [30], we are unaware of any studies linking HMGB3 and breast cancer.

In summary, miR-205 is an important tumor suppressor in breast cancer. It has been shown to regulate a number of important oncogenic targets including ZEB1, VEGFA and HER3 reviewed in 16. Our data suggest that in addition to HMGB3, miR-205 may modulate a several additional targets such as PLXNA4 and RUNX2 (Figure 3C). It is possible therefore that HMGB3 and other oncogenic proteins may be reduced in advanced breast cancer via miR-205 derepression using epigenetic modifying drugs. Of note is the fact that 5-Aza is currently in phase II clinical trials for advanced breast cancer (ClinicalTrials.gov). Modulation of downstream targets of miR-205 such as HMGB3, through miR-205 derepression may enhance our understanding of the therapeutic benefit of these drugs.

## Supporting Information

Figure S1 **N-cadherin, Vimentin, ZEB1 and β-actin protein levels were determined by western blotting in BT549 cells 48 hr after transfection of pre-miR-negative or pre-miR-205.** Protein was harvested using CellLytic™ MT (Sigma Aldrich, St. Louis, MO) and 1X protease and phosphatase inhibitor (Pierce, Rockford, IL) using standard techniques. Normalized expression to β-actin is indicated at each concentration condition.
(TIF)

Figure S2 (A) qPCR verification of relative expression levels of CDH1 in MDA-MB-231 cells after transfection of HMGB3 siRNA. Individual mRNA expression levels were calculated relative to 18S rRNA and the data are expressed as fold change under input control, which was assigned a value of “1”. (B) MDA-MB-231 cells were transfected with control siRNA or HMGB3 siRNA for 48 hours. HMGB3, CDH1 and ZEB1 proteins were measured by immunoblotting in MDA-MB-231 cells transfected with control siRNA or HMGB3 siRNA. GAPDH served as a loading control. Relative intensities are indicated above each band.
(TIF)

Table S1 **Tissue information.**
(XLSX)

Table S2 **Primer sequences.**
(XLSX)

Methods S1 (DOCX)
