# Supplementary figures and images for: Tumor Suppressive Function of mir-205 in Breast Cancer Is Linked to HMGB3 Regulation
Source: PLoS One. 2013 Oct 2;8(10):e76402. doi: 10.1371/journal.pone.0076402 (PMC3788717; doi:10.1371/journal.pone.0076402)

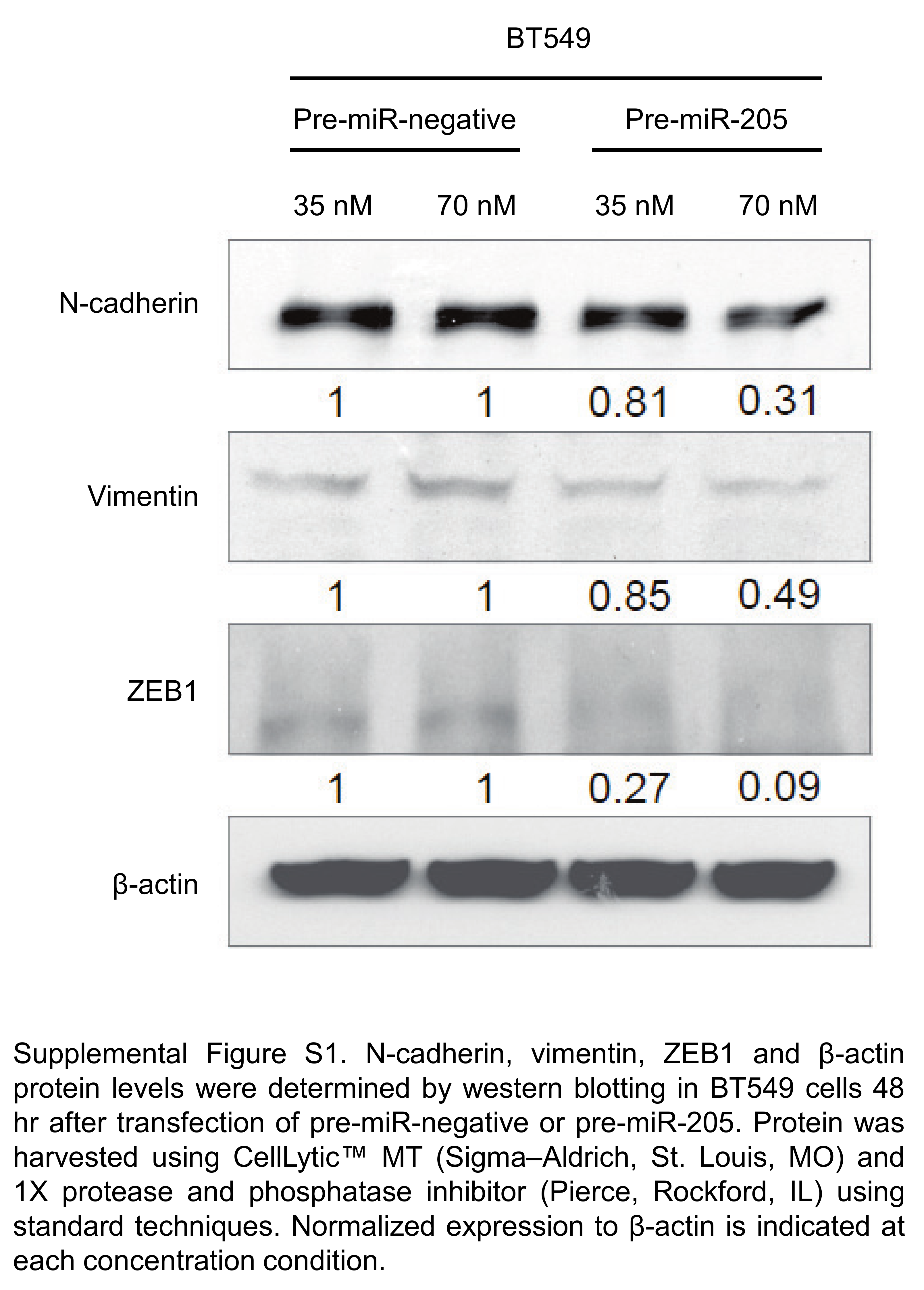

Supplement: Figure S1 — N-cadherin, Vimentin, ZEB1 and β-actin protein levels were determined by western blotting in BT549 cells 48 hr after transfection of pre-miR-negative or pre-miR-205. Protein was harvested using CellLytic™ MT (Sigma Aldrich, St. Louis, MO) and 1X protease and phosphatase inhibitor (Pierce, Rockford, IL) using standard techniques. Normalized expression to β-actin is indicated at each concentration condition. (TIF) [file pone.0076402.s001.tif]

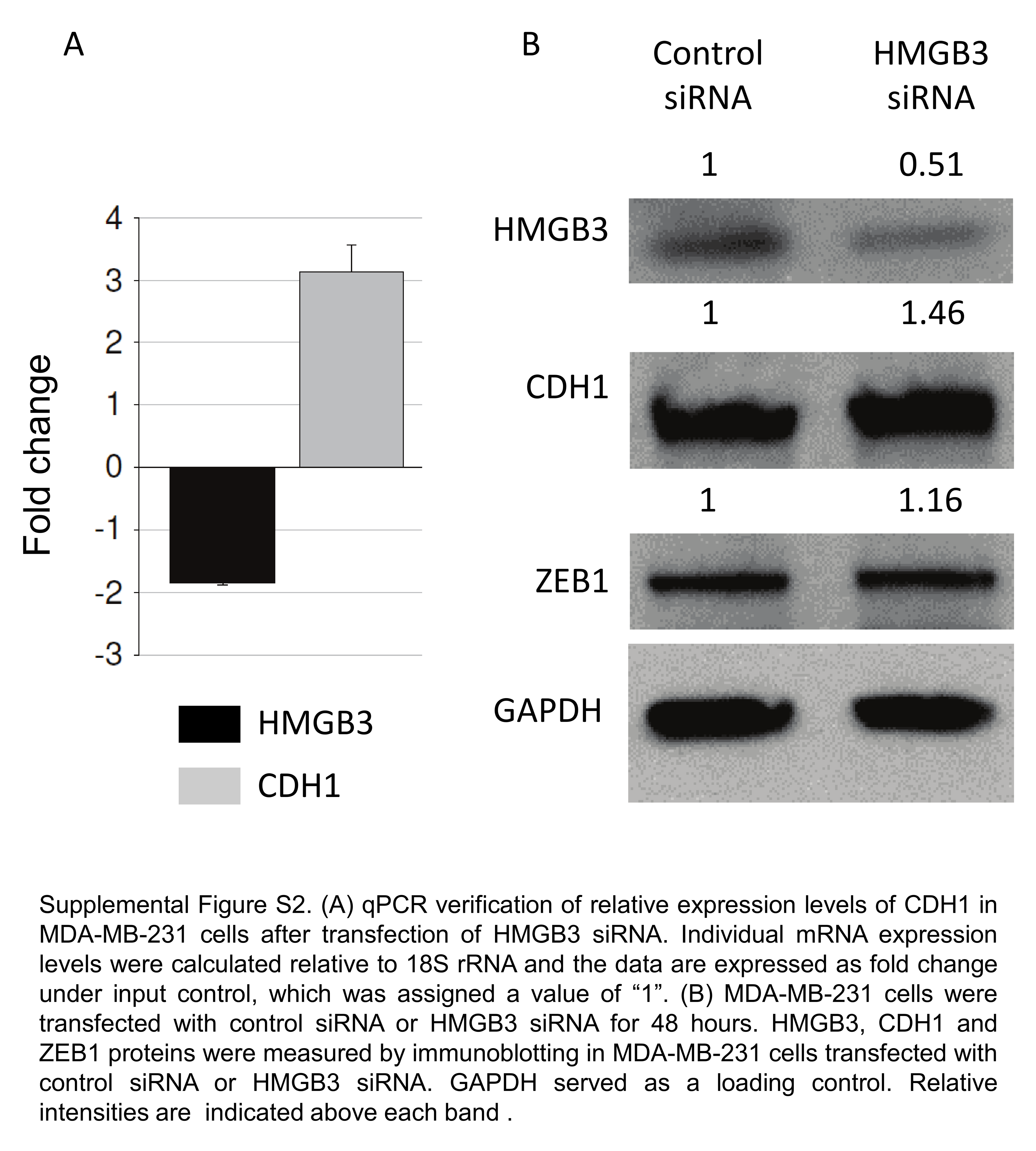

Supplement: Figure S2 — (A) qPCR verification of relative expression levels of CDH1 in MDA-MB-231 cells after transfection of HMGB3 siRNA. Individual mRNA expression levels were calculated relative to 18S rRNA and the data are expressed as fold change under input control, which was assigned a value of “1”. (B) MDA-MB-231 cells were transfected with control siRNA or HMGB3 siRNA for 48 hours. HMGB3, CDH1 and ZEB1 proteins were measured by immunoblotting in MDA-MB-231 cells transfected with control siRNA or HMGB3 siRNA. GAPDH served as a loading control. Relative intensities are indicated above each band. (TIF) [file pone.0076402.s002.tif]
