# Supplementary material for: Tumor Suppressive Function of mir-205 in Breast Cancer Is Linked to HMGB3 Regulation
Source: PLoS One. 2013 Oct 2;8(10):e76402. doi: 10.1371/journal.pone.0076402 (PMC3788717; doi:10.1371/journal.pone.0076402)
Supplement: Methods S1 — (DOCX) [file pone.0076402.s005.docx]

Methods:

Kaplan Meyer survival plot.

A disease free survival for breast cancer patients was analyzed as a function of HMGB3 gene expression using open access web resource G-DOC developed at Georgetown University Medical Center [1].

The G-DOC data repository is designed to store multiple types of metadata associated with individual samples and patients including demographic data, clinical outcome, and tumor-specific phenotype data as well as molecular profiling data such as gene and microRNA expression. The data in G-DOC are uniformly processed using validated algorithms within the R-based bioinformatics toolbox (Bioconductor) [2], formatted and mapped using R scripts, and then uploaded to the central database. Current version of G-DOC 2.0 contains 26 breast cancer studies with total number of 3653 patients. To assess a role of HMGB3 gene expression in clinical outcome of breast cancer we have analyzed a dataset from Loi et al study [3] using KM survival analysis tools that are build-in in G-DOC web portal [1]. Log ratios of gene expression was used to construct KM for disease free survival of breast cancer patients. 3 groups of patients were compared: Patients with HMGB3 gene down-regulated by more than 1.2 fold; Patients with genes overexpressed by more than 1.2 fold; Patients with intermediate values of fold change. A significance of differences between each pair of KM curves was estimated based on p-values of logrank test [4]. The breast cancer studies and the analysis tools are freely available via open access webportal at gdoc.georgetown.edu.

References:

1. Madhavan S, Gusev Y, Harris M, Tanenbaum DM, Gauba R, Bhuvaneshwar K, Shinohara A, Rosso K, Carabet LA, Song L, Riggins RB, Dakshanamurthy S, Wang Y, Byers SW, Clarke R, Weiner LM. G-DOC: A Systems Medicine Platform for Personalized Oncology. Neoplasia 2011 13(9): 771–783

2. Gentleman RC, Carey VJ, Bates DM, Bolstad B, Dettling M, Dudoit S, Ellis B,

Gautier L, Ge Y, Gentry J, et al. Bioconductor: open software development for computational biology and bioinformatics. Genome Biol 2004 5(10), R80

3. Loi S, Haibe-Kains B, Desmedt C, Wirapati P, Lallemand F, Tutt AM, Gillet C, Ellis P, Ryder K, Reid JF, Daidone MG, Pierotti MA, Berns EM, Jansen MP, Foekens JA, Delorenzi M, Bontempi G, Piccart MJ, Sotiriou C. Predicting prognosis using molecular profiling in estrogen receptor–positive breast cancer treated with tamoxifen. BMC Genomics. 2008 9:239

4. Mantel N . Evaluation of survival data and two new rank order statistics arising in its consideration. Cancer Chemotherapy Reports 1966 50 (3): 163–70
